# Solid-phase extraction of palladium, platinum, and gold from water samples: comparison between a chelating resin and a chelating fiber with ethylenediamine groups

**DOI:** 10.1007/s44211-023-00270-3

**Published:** 2023-01-19

**Authors:** Misato Iwase, Kota Isobe, Linjie Zheng, Shotaro Takano, Yoshiki Sohrin

**Affiliations:** Institute for Chemical Research, Kyoto University, Uji, Kyoto 611-0011 Japan

**Keywords:** Palladium, Platinum, Gold, Solid-phase extraction, Chelating resin, Chelating fiber

## Abstract

Dissolved palladium (Pd), platinum (Pt), and gold (Au) form inert chloride complexes at low concentrations of pmol/kg in environmental water, thus rendering difficulty in the development of a precise analytical method for these metals. Herein, we report the preconcentration of Pd, Pt, and Au with a chelating fiber Vonnel-en and a chelating resin TYP-en with ethylenediamine (en) groups. Batch adsorption experiments reveal the adsorption capacity of Vonnel-en for Pd(II), Pt(IV), and Au(III) in 0.10 M HCl as 0.53, 0.22, and 0.27 mmol/g, respectively. The adsorption capacity of TYP-en for Pd(II), Pt(IV), and Au(III) in 0.10 M HCl is 0.31, 0.17, and 0.52 mmol/g, respectively. In column extraction experiments using small-volume samples containing Pd(II), Pt(II), Pt(IV), Au(I), or Au(III) at concentrations of μmol/kg, TYP-en is able to quantitatively recover Pd, Pt, and Au from 0.01 to 0.2 M HCl irrespective of their oxidation states. In contrast, Vonnel-en is unable to quantitatively recover Au(I). In column extraction experiments using large-volume samples containing Pd(II), Pt(IV), and Au(III) at concentrations of pmol/kg, the recovery of Pd(II), Pt(IV), and Au(III) by TYP-en from 0.07 M HCl is 100–105%. However, the recovery of Pd(II), Pt(IV), and Au(III) by Vonnel-en from 0.03 to 0.3 M HCl is 102–110, 7–15, and 20–52%, respectively. Thus, the chelating resin TYP-en has a high potential for the multielemental determination of Pd, Pt, and Au in environmental water.

**Graphical abstract:**

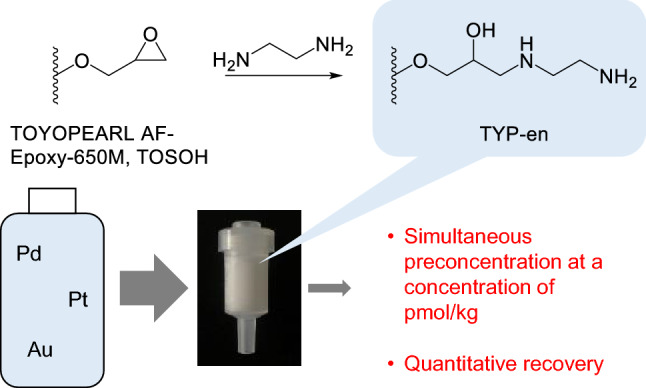

**Supplementary Information:**

The online version contains supplementary material available at 10.1007/s44211-023-00270-3.

## Introduction

Pd, Pt, and Au are precious metals with special properties, such as high electrical conductivity, resistance to corrosion, high melting point, and catalytic ability. Pd is used in chemical catalysts, dental applications, electronic appliances, and jewelry [1, 2]. Pt is used in electronic appliances, chemical catalysts, jewelry, and medical applications [2]. Moreover, Pd and Pt are used predominantly in the three-way catalysts of gasoline vehicles [1, 2], whereas Au is used primarily in industrial fields, such as electronic and mechanical, and is also used in dental applications and jewelry [3].

Pd, Pt, and Au are siderophile elements, and their abundance in the earth’s crust is extremely low, at 0.4, 0.4, and 2.5 ng/g, respectively [4]. The Pd and Pt cycles on the Earth’s surface are affected by anthropogenic activities [5]. Pd and Pt concentrations in Greenland snow has increased by approximately 60–100-fold compared with that in ice cores of 7000 years ago [6]. In addition, Pd and Pt concentrations in airborne particulate matter and in soil of urban areas are higher than those of rural areas [7, 8]. High concentrations of Pt are observed in hospital sewage owing to Pt-based anticancer drugs for anticancer chemotherapy [9]. High concentrations of Au are found in urban sewage sludge, which suggests anthropogenic sources [10]. These precious metals are transported to the aquatic environment and bioaccumulated to aquatic organisms via the food chain [1, 11]. In addition, it was reported that certain Pt compounds are cytotoxic and have mutagenic and carcinogenic effects [11]. Gold nanoparticles of 1–2 nm in size are highly cytotoxic [12]. Although little is known about Pd toxicity, increased emission of the precious metals may become a threat to the sustainability of aquatic ecosystems. Therefore, the distribution of Pd, Pt, and Au in the aquatic environment must be observed to achieve SDGs.

These precious metals are present at very low concentrations (pmol/kg) in environmental waters. The oxidation states are as Pd(II), Pt(II), Pt(IV), Au(I), and Au(III) [13, 14]. These ions form inert complexes with chloride ions: Pd${\text{Cl}}_{4}^{2-}$, Pt${\text{Cl}}_{4}^{2-}$, Pt${\text{Cl}}_{6}^{2-}$, Pt${\text{Cl}}_{5}{\text{(OH)}}^{2-}$, Au${\text{Cl}}_{2}^{-}$, and Au${\text{Cl}}_{3}{\text{(OH)}}^{-}$ [13, 15]. Thus, the determination of Pd, Pt, and Au in environmental waters is a challenge in analytical chemistry [2]. Conventionally, ion exchange methods have been used for the preconcentration of these metals [16–19]. Anion exchange resins, AG1-X8 [20, 21] or Dowex^™^ 1-X8 [22], have been used to determine the concentrations of Pd and Pt in river water and seawater. In addition, some chelating resins have been used for the preconcentration of Pd, Pt, and Au; these include the Nobias Chelate-PA1^®^ [23], Presep^®^ PolyChelate [24], and MetaSEP AnaLig PM-05 [25]. However, these methods have drawbacks, such as a low recovery percentage. In addition, the simultaneous preconcentration of Pd, Pt, and Au at concentrations of pmol/kg has not been extensively examined. It has been reported that column extraction using a chelating fiber, poly(*N*-aminoethyl)acrylamide with ethylenediamine (en) groups, has the potential to quantitatively recover Au(III), Pt(IV), Pd(IV), Ir(IV), Ru(III), and Rh(III) at concentrations of nmol/kg [26]. It was also reported that chelating fiber synthesized by amination of acrylic fiber acts as an effective adsorbent for Cr(III), Cr(VI), Cu(II), and As(V) [27, 28]. The ultimate goal of the present study is to develop a novel analytical method that can simultaneously determine Pd, Pt, and Au at a concentration of pmol/kg. As the first step, we prepared a chelating fiber and a chelating resin with en groups using the synthetic method of poly(*N*-aminoethyl)acrylamide [26]. Herein, we report the results of both the batch and column methods of solid-phase extraction for Pd, Pt, and Au using chelating adsorbents with en groups in order to make the difference between fiber and resin clear.

## Experimental

### Reagents and materials

Reagent-grade HCl, HNO_3_, NH_3_, H_2_O_2_, methanol, acetone, en, and NaOH were obtained from FUJIFILM Wako Pure Chemical (Japan). EMSURE^®^-grade KCN was obtained from Merck (Germany). A sample of acrylic staple fiber, Vonnel^™^, was obtained from Mitsubishi Chemical (Japan). TOYOPEARL AF-Epoxy-650 M (particle size, 40–90 μm; amount of epoxy group, 0.8 mmol/g) was obtained from TOSOH (Japan). Standard solutions of Pd(II), Pt(IV), and Au(III) were prepared from 1000 mg/ml standard solutions (Wako Pure Chemicals). Wako 1st grade K_2_[PtCl_4_] and practical grade K[Au(CN)_2_] were obtained from Wako Pure Chemical to prepare the standard solutions of Pt(II) and Au(I), respectively. Deionized water (MQW) purified using a Milli-Q IQ 7005 system (Merck) was used throughout the experiments. We paid special attention for eluents of NH_3_–KCN and HCl–H_2_O_2_ during preparation and usage. We did not acidify NH_3_–KCN solution to avoid emission of HCN. We treated HCl–H_2_O_2_ solution in a fume hood to avoid inhalation of Cl_2_.

Nalgene low-density polyethylene (LDPE) bottles (Thermo Fisher Scientific) were used to store the solutions. Polypropylene tubes (SARSTEDT, Germany) were used for batch adsorption experiments. Nalgene perfluoroalkoxy alkane (PFA) bottles (Thermo Fisher Scientific, USA) were used for evaporation on a hot plate (AS ONE, Japan). The bottles and tubes were soaked overnight in an alkaline detergent (5% Scat 20-X, Nacalai Tesque, Japan), rinsed with tap water, soaked overnight in 4 M HCl, and subsequently rinsed with MQW. Finally, they were soaked overnight in 0.3 M NH_3_–0.03 M KCN and rinsed with MQW.

### Apparatus

The elemental concentrations in the range of μmol/kg were determined by a calibration curve method using SPECTROBLUE ICP-AES (SPECTRO, Germany). Elemental concentrations ranging from nmol/kg to pmol/kg were determined by a calibration curve method using a NexION 350D quadrupole ICP-MS (Perkin Elmer, USA). Sample solutions were introduced into the NexION using a desolvating nebulizer Apex Q (Elemental Scientific, USA). The measured mass numbers were 105 and 108 for Pd, 194 and 195 for Pt, and 197 for Au.

### Synthesis of Vonnel-en

**Fig. 1:**
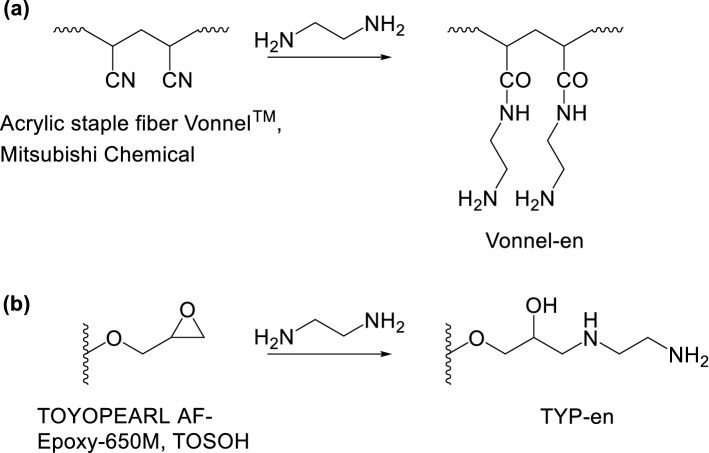
Synthetic reactions of **a** Vonnel-en and **b** TYP-en

A sample of acrylic staple fiber Vonnel^™^ was cut into approximately 5 mm-long pieces and soaked in acetone overnight. Subsequently, the fiber was soaked in methanol overnight, rinsed with MQW, and dried at 50 °C in a drying oven (Yamato Scientific, Japan). A 2.5 g portion of the dried fiber and 125 g of 9 M en solution were mixed at pH 12.5 in an LDPE bottle and shaken at 260 rpm and 70 °C for 15 h using a constant-temperature incubator shaker (TAITEC, Japan). The reaction is shown in Fig. 1a. The Vonnel-en fiber was washed with MQW until the wash solution attained a neutral pH. Subsequently, it was dried at 50 °C in a drying oven and stored in an LDPE bottle in a desiccator.

### Synthesis of TYP-en

A 2.4 g portion of TOYOPEARL AF-Epoxy-650 M and 144 g of 200 mM en solution were mixed in an LDPE bottle and the pH was adjusted to pH 11 by adding 1 M HCl. The mixture was shaken at 260 rpm and 70 °C for 36 h in a constant-temperature incubator shaker. The reaction is shown in Fig. 1b. The TYP-en resin was sieved using a Teflon screen with 170 mesh to remove fine particles. The TYP-en resin was washed with MQW until the wash solution became neutral and stored in MQW in an LDPE bottle.

### Batch adsorption experiments

In a 50 ml polypropylene tube, 25 g of a sample solution containing 40–500 μmol/kg of a metal ion was mixed with 20 mg of Vonnel-en or TYP-en and agitated at 200 rpm and 25 °C for 2 h using a constant-temperature incubator shaker. Subsequently, the adsorbent was collected on a 0.2 μm Nuclepore membrane and dried at 50 °C in a drying oven. The dry weight of the adsorbent was determined using a balance (SHIMADZU, Japan). The concentration of the metal ions in the filtrate was determined using ICP-AES. The amount of metal ions adsorbed on the adsorbent was assumed to be the difference between the metal ion amounts in the sample solution and the filtrate. The adsorption capacity of the metal ions is calculated using the following equation:

$${\text{Adsorption capacity }}\left( {\text{mmol/g}} \right) \, = \, \frac{{(C_{{\text{s}}} \, - \, C_{{\text{f}}} ) \, \times \, W_{{\text{s}}} }}{{W_{{\text{a}}} }}$$

where *C*_s_ and *C*_f_ represent the concentrations of the metal ions in the sample solution and filtrate, respectively; and *W*_s_ and *W*_a_ represent the weights of the sample solution and the dried adsorbent, respectively.

### Column extraction experiments

The Vonnel-en fiber (approximately 100 mg dry weight) or the TYP-en resin (approximately 200 mg dry weight) was packed in a Type-M cartridge column (TOMOE, Japan), with a polypropylene body and polyethylene frits, with an inner diameter of 8.4 mm and a bed height of 8.5 mm.

**Fig. 2:**
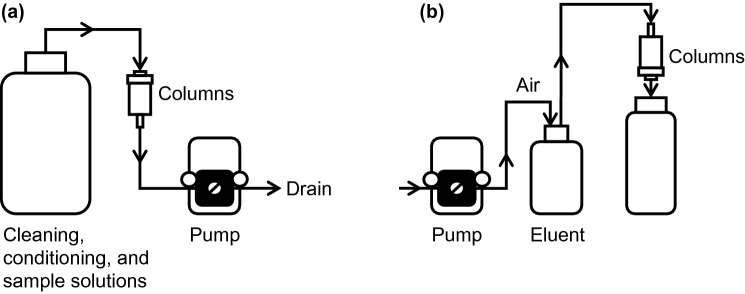
Diagrams of **a** preconcentration system and **b** elution system

The column experiments were performed in a clean hood. Prior to use, the column was cleaned by passing successively 500 ml of 0.3 M NH_3_–0.03 M KCN at a flow rate of 0.2 ml/min and 100 ml of MQW at a flow rate of 2 ml/min. A preconcentration system was constructed with the column, LDPE bottles, PFA tubes with an internal diameter of 3 mm, Tygon tubes (SAINT-GOBAIN, France) with that of 3.18 mm, PharMed tubes (TOKYO RIKAKIKAI, Japan) with that of 2.15 mm, and a cassette tube pump SMP-23 (TOKYO RIKAKIKAI) (Fig. 2a). A column of Vonnel-en or TYP-en was conditioned by successively passing 20 ml of 0.1 M NH_3_, 20 ml of MQW, and 20 ml of HCl solution with the same HCl concentration as the sample solution at a flow rate of 2 ml/min just before column extraction. For column extraction experiments using a small-volume sample containing a metal ion at a high concentration, the samples used were: 25 g of 0.01–0.2 M HCl solutions containing 50 μmol/kg of Pd(II) or 25 μmol/kg of Pt(II), Pt(IV), Au(I) or Au(III). For column extraction experiments using a large-volume sample containing metal ions at low concentrations, the samples used were: 500 g of 0.03–0.3 M HCl solutions containing 35 pmol/kg of Pd(II), Pt(IV), and Au(III) or 600 g of 0.03–0.3 M HCl solutions containing 50 pmol/kg of Pd(II), Pt(IV), and Au(III). The sample solution was loaded into the column at a flow rate of 1 ml/min. The solution that passed through the column during the sample loading was collected in an LDPE bottle. After sample loading, 20 ml of HCl solution with the same HCl concentration as the sample solution was passed through the column at a flow rate of 2 ml/min to remove remaining salts from sample solution.

Subsequently, the column was detached from the preconcentration system and attached to an elution system, which was constructed with the column, LDPE bottles, PFA tubes with an internal diameter of 2 mm, Tygon tubes with that of 3.18 mm, PharMed tubes with that of 1.15 mm, and a cassette tube pump SMP-23 (Fig. 2b). The elution system flowed through the column in the direction opposite to that of the preconcentration system. Pd, Pt, and Au adsorbed on Vonnel-en or TYP-en were eluted with 0.3 M NH_3_–0.003 M KCN or 8 M HCl–10 mM H_2_O_2_ at a flow rate of 0.6 ml/min. When the metal ions remained in the column after elution with 8 M HCl–10 mM H_2_O_2_, they were further eluted with 0.3 M NH_3_–0.003 M KCN. Subsequently, the eluates were collected in PFA bottles. The column was again mounted on the preconcentration system and cleaned with 30 ml of MQW at a flow rate of 2 ml/min.

The eluate of 0.3 M NH_3_–0.003 M KCN was directly introduced to ICP-AES or ICP-MS to measure Pd, Pt, and Au. The eluate of 8 M HCl–10 mM H_2_O_2_, collected in a PFA bottle, was evaporated to dryness for 8 h at 180 °C on a hot plate. After evaporation, the residue was re-dissolved in 3 ml of 1 M HCl–1 M HNO_3_ at 270 rpm and 70 °C for 8 h in a constant-temperature incubator. The re-dissolved solution was used for measurement. The adsorption and recovery percentages are calculated using the following equations:

$${\text{Percentage of adsorption }}\left( \% \right) \, = \, \frac{{C_{{\text{s}}} \, - \, C_{{\text{p}}} }}{{C_{{\text{s}}} }} \, \times \, 100$$

$${\text{Percentage of recovery }}\left( \% \right) \, = \, \frac{{C_{{\text{e}}} \, \times \, W_{{\text{e}}} }}{{C_{{\text{s}}} \, \times \, W_{{\text{s}}} }} \, \times \, 100$$

where *C*_s_, *C*_p_, and *C*_e_ represent the concentration of metal ions in the sample solution, the solution passing through the column during sample loading, and the eluate, respectively; and *W*_s_ and *W*_e_ represent the weights of the sample solution and the eluate, respectively. When evaporation–redissolution was applied, the concentration and weight of the re-dissolved solution were used instead of *C*_e_ and *W*_e_.

## Results and discussion

### Adsorption and elution conditions

**Fig. 3:**
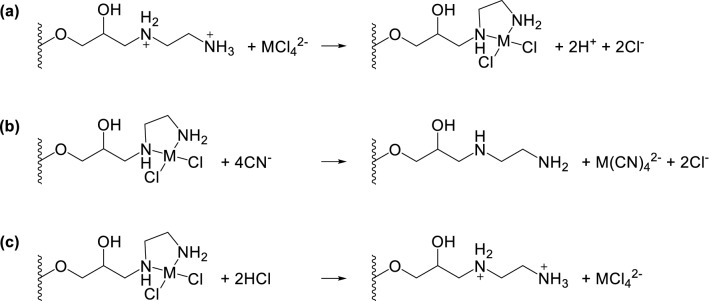
**a** Adsorption reaction of metal ions on TYP-en from HCl solution. Elution reaction of metal ions in **b** NH_3_–KCN solution and **c** HCl–H_2_O_2_ solution

Pd, Pt, and Au form negatively charged chloride complexes in HCl solution [13–15]. TYP-en and Vonnel-en have positive charges in HCl solution owing to protonation of the en groups. Therefore, the negatively charged Pd, Pt, and Au chloride complexes are attracted and adsorbed in the adsorbent through electrostatic forces, thereby forming chelates (Fig. 3a).

The preliminary experiments revealed that the good eluents for Pd, Pt, and Au were 0.3 M NH_3_–0.003 M KCN and 8 M HCl–10 mM H_2_O_2_ (Supplementary Figs. S1–S3). In 0.3 M NH_3_–0.003 M KCN, Pd, Pt, and Au formed negatively charged cyanide complexes (Fig. 3b). As the en groups were neutral in this solution, the cyanide complexes were easily removed. Although this eluent was strong, some defects were observed: (1) cyanide ions are highly toxic; (2) potassium ions interfere with the determination of Pd, Pt, and Au by ICP-MS; and (3) when this eluent was applied to natural samples, some ferric ions that were adsorbed on the adsorbent from the sample solution formed iron hydroxide during elution, which adsorbed Pd, Pt, and Au, thereby resulting in a low recovery in the eluate (Supplementary Table S1).

In 8 M HCl–10 mM H_2_O_2_, reverse adsorption reaction occurred owing to the high concentration of chloride ions (Fig. 3c). This eluent was not very strong owing to the electrostatic forces between the chloride complex ions and the adsorbent, and a large volume of the eluent was necessary for quantitative recovery. However, the defects of 0.3 M NH_3_–0.003 M KCN were overcome. When 8 M HCl was used as the eluent, Au recovery was not quantitative. We assumed that this was caused by the formation of Au(0) on the adsorbent due to the following disproportionation reaction:

$$3{\text{AuCl}}_{2}^{ - } {\mkern 1mu} \rightleftharpoons {\text{ AuCl}}_{4}^{ - } {\mkern 1mu} + {\mkern 1mu} 2{\text{Au}}\left( 0 \right){\mkern 1mu} + {\mkern 1mu} 2{\text{Cl}}^{ - }$$

We added H_2_O_2_ to 8 M HCl to oxidize Au(0) using the following reaction:

$$2{\text{Au(0)}}{\mkern 1mu} + {\mkern 1mu} 3{\text{H}}_{{2}} {\text{O}}_{{2}} {\mkern 1mu} + {\mkern 1mu} 8{\text{HCl }} \to \, {\mkern 1mu} 2{\text{AuCl}}_{{4}}^{ - } {\mkern 1mu} + {\mkern 1mu} 2{\text{H}}^{ + } {\mkern 1mu} + {\mkern 1mu} 6{\text{H}}_{{2}} {\text{O}}$$

Evidently, Au recovery was higher in the 8 M HCl–10 mM H_2_O_2_ solution. Therefore, it was considered as the most promising eluent.

### Adsorption capacity

**Fig. 4:**
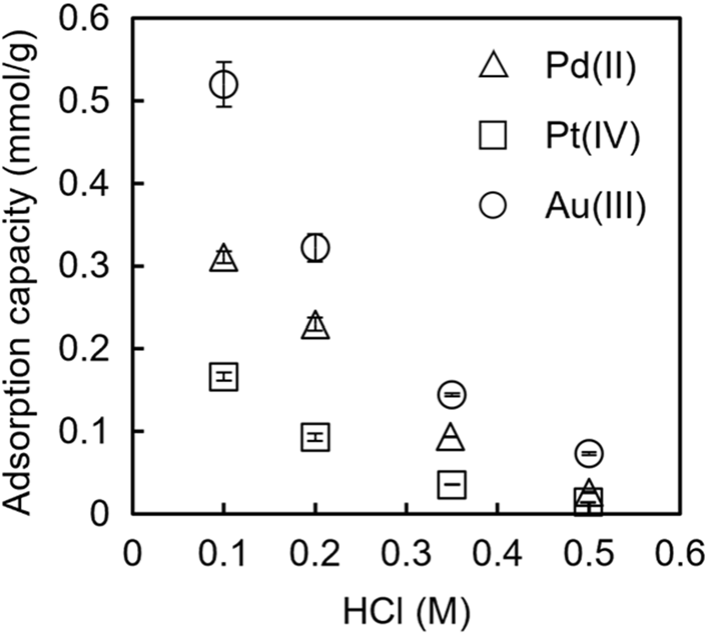
Effect of HCl concentration on the adsorption capacity of TYP-en for Pd(II), Pt(IV), and Au(III). Metal ion concentration in sample solution: 100–500 μmol/kg Pd(II), 40–200 μmol/kg Pt(IV), or 40–400 μmol/kg Au(III). Error bars show the standard deviation (*n* = 2)

First, using TYP-en, the HCl concentration dependency of the adsorption capacities of Pd(II), Pt(IV), and Au(III) were examined (Fig. 4). We repeated the experiments for each condition and obtained highly reproducible data. Evidently, the adsorption capacities of Pd(II), Pt(IV), and Au(III) decreased when the HCl concentration increased from 0.1 M to 0.5 M. Subsequently, the adsorption capacities of Pd(II), Pt(IV), and Au(III) in 0.10 M HCl between Vonnel-en and TYP-en were compared (Table 1). The adsorption capacities of Pd(II) and Pt(IV) for Vonnel-en were 1.7 and 1.3 times higher than those for TYP-en, respectively. In contrast, the adsorption capacity of TYP-en for Au(III) was 1.9 times higher than that for Vonnel-en. The reason of these opposite results is not clear now. Vonnel^™^ is copolymer of acrylamide and other constituents, of which detail is not open to the public. It is possible that the other constituents influence the adsorption behavior of Vonnel-en. As approximately 100 mg dry weight of the Vonnel-en fiber or approximately 200 mg dry weight of the TYP-en resin was packed in a column, the adsorption capacity of the column was higher than 0.022 mmol for Pd(II), Pt(IV), and Au(III), which was > 35 times higher than the metal ion amount in the subsequent column extraction experiments.

**Table 1:** Adsorption capacities of Vonnel-en and TYP-en

Element	HCl (M)	Adsorption capacity (mmol/g)			
		Vonnel-en		TYP-en	
		*n*	ave ± sd	*n*	ave ± sd
Pd(II)	0.10	3	0.526 ± 0.006	2	0.310 ± 0.007
Pt(IV)	0.10	3	0.217 ± 0.004	2	0.166 ± 0.005
Au(III)	0.10	3	0.27 ± 0.02	2	0.52 ± 0.03

### Column extraction experiments using small-volume samples containing a metal ion at a high concentration

**Fig. 5:**
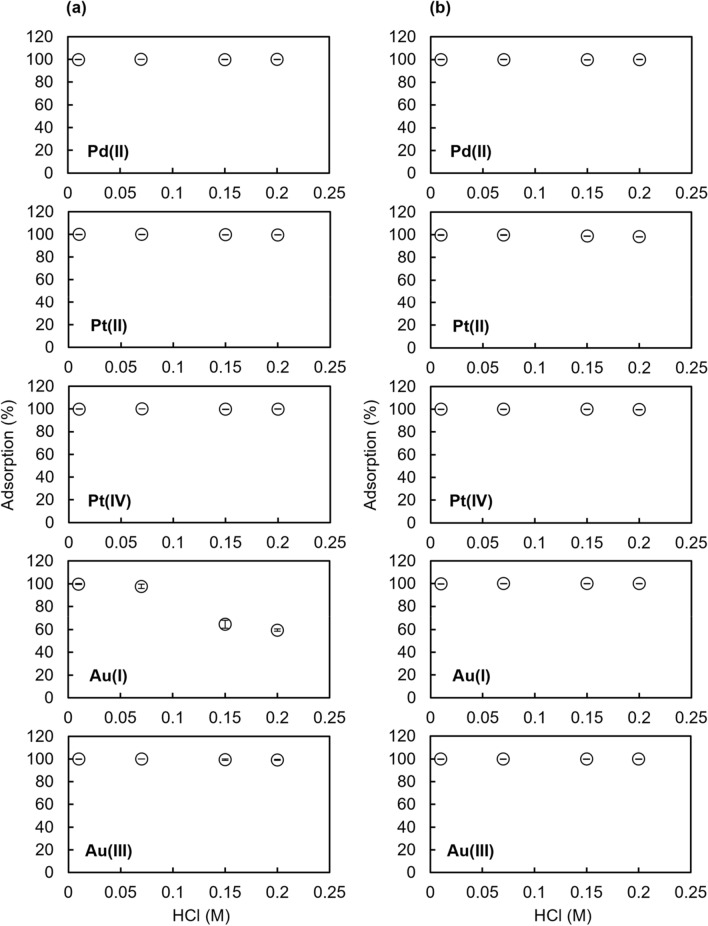
Effect of HCl concentration on the adsorption percentage of Pd(II), Pt(II), Pt(IV), Au(I), and Au(III) for **a** Vonnel-en and **b** TYP-en. Metal ion concentration in sample solution: 50 μmol/kg Pd(II) or 25 μmol/kg Pt(II), Pt(IV), Au(I), or Au(III). Error bars show the standard deviation (*n* = 3)

The effect of HCl concentration on the percentage of adsorption for Vonnel-en and TYP-en in column extraction experiments was investigated using small-volume samples of 25 g containing a metal ion at a high concentration of 25–50 μmol/kg (Fig. 5). For Vonnel-en, Au(I) was not quantitatively collected from > 0.15 M HCl solution. In contrast, TYP-en was able to quantitatively recover Pd(II), Pt(II), Pt(IV), Au(I), and Au(III) from 0.01 to 0.2 M HCl solution.

**Fig. 6:**
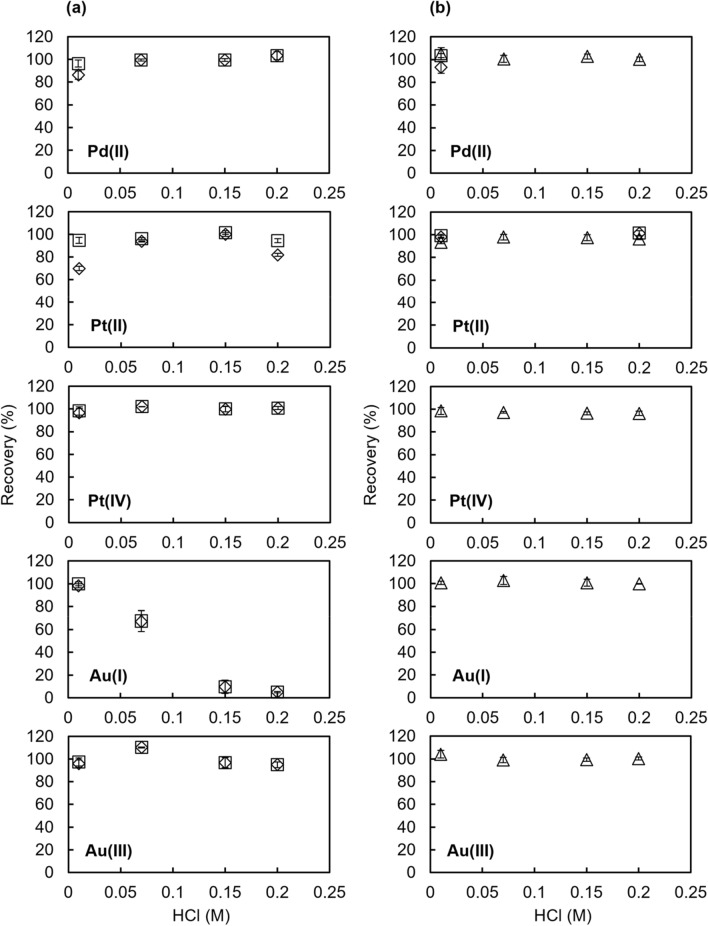
Effect of HCl concentration on the recovery percentage of Pd(II), Pt(II), Pt(IV), Au(I), and Au(III) for **a** Vonnel-en and **b** TYP-en. Metal ion concentration in sample solution: 50 μmol/kg Pd(II) or 25 μmol/kg Pt(II), Pt(IV), Au(I), or Au(III). Eluent: 90 g of 0.3 M NH_3_–0.003 M KCN (triangles), 90 g of 8 M HCl–10 mM H_2_O_2_ (diamonds), or 90 g of 8 M HCl–10 mM H_2_O_2_ and 30 g of 0.3 M NH_3_–0.003 M KCN (squares). Error bars show the standard deviation (*n* = 3)

In addition, the effect of the HCl concentration on the recovery of Vonnel-en and TYP-en was investigated (Fig. 6). Pd(II), Pt(IV), and Au(III) were quantitatively recovered from a 0.07–0.2 M HCl solution by both Vonnel-en and TYP-en. Pt(II) recovery by Vonnel-en was 70–100% when the eluent was 8 M HCl–10 mM H_2_O_2_. However, a quantitative recovery was obtained by additional elution with 0.3 M NH_3_–0.003 M KCN. Au(I) recovery by Vonnel-en decreased with increasing HCl concentration, and the obtained recoveries from 0.07 to 0.2 M HCl solution were lower than the percentages of adsorption. This was because a portion of Au(I) adsorbed on the Vonnel-en was desorbed when 20 ml of HCl solution with the same concentration as the sample solution was passed through the column after sample loading. In contrast, Pt(II) and Au(I) were quantitatively recovered from 0.01 to 0.2 M HCl solution by TYP-en with an eluent of 0.3 M NH_3_–0.003 M KCN or 8 M HCl–10 mM H_2_O_2_. Thus, TYP-en is expected to have the potential to determine the total dissolved concentrations of Pd, Pt, and Au irrespective of their oxidation states.

### Column extraction experiments using large-volume samples containing metal ions at low concentrations

**Fig. 7:**
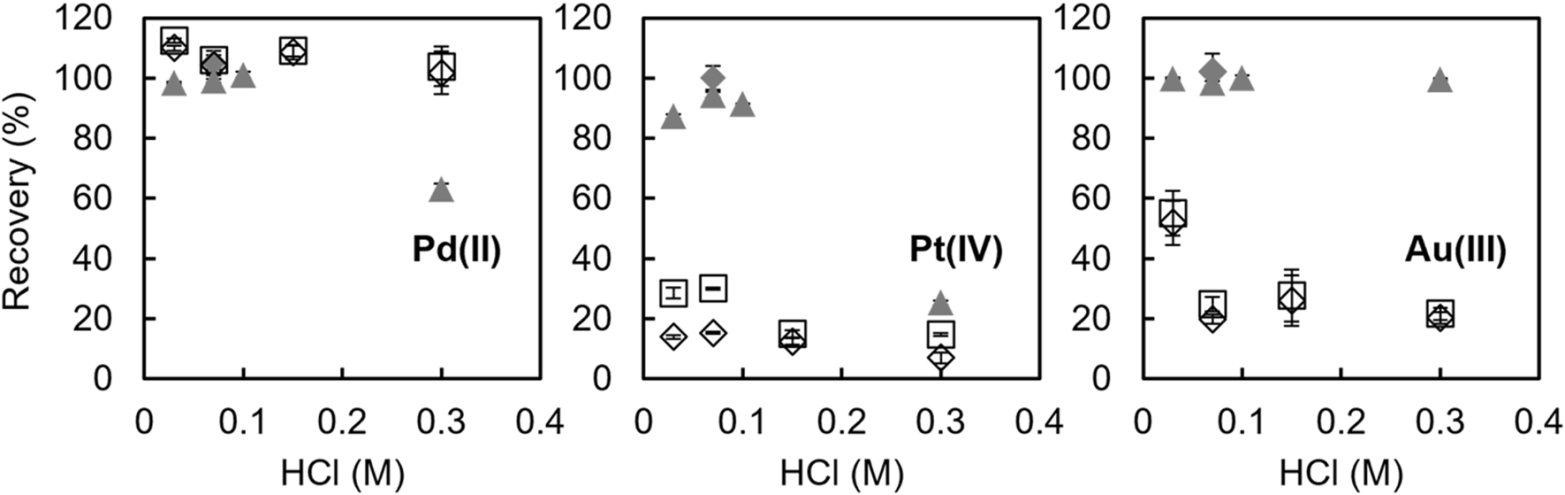
Effect of HCl concentration on the recovery percentage of Pd(II), Pt(IV), and Au(III) for Vonnel-en (white symbols) and TYP-en (gray symbols). Sample solution: 500 g of 0.03–0.3 M HCl containing 35 pmol/kg Pd(II), Pt(IV), and Au(III) (white diamonds, white squares, and gray diamonds) or 600 g of 0.03–0.3 M HCl containing 50 pmol/kg Pd(II), Pt(IV), and Au(III) (gray triangles). Eluent: 60 g of 0.3 M NH_3_–0.003 M KCN (triangles), 180 g of 8 M HCl–10 mM H_2_O_2_ (diamonds), or 180 g of 8 M HCl–10 mM H_2_O_2_ and 30 g of 0.3 M NH_3_–0.003 M KCN (squares). Error bars show the standard deviation (*n* = 3)

Column extraction experiments were performed using large-volume samples (500–600 g) containing Pd(II), Pt(IV), and Au(III) at a low concentration of 35–50 pmol/kg (Fig. 7). Pd(II) was quantitatively recovered from 0.03 to 0.3 M HCl solution by Vonnel-en and from 0.03 to 0.1 M HCl solution by TYP-en. Pt(IV) was quantitatively recovered from 0.07 M HCl solution and Au(III) was quantitatively recovered from 0.03 to 0.3 M HCl solution by TYP-en. The recovery of Pt(IV) and Au(III) from 0.03 to 0.3 M HCl solution by Vonnel-en was 7–15% and 20–52%, respectively, when the eluent was 8 M HCl–10 mM H_2_O_2_. Additional elution with 0.3 M NH_3_–0.003 M KCN slightly increased the recovery of Pt(IV) and Au(III) to 15–30% and 22–55%, respectively. These results indicate that Pt(IV) and Au(III) were not quantitatively retained by Vonnel-en, although the total amount of metal ions was only 53 pmol in these experiments. Thus, the recovery of Pt(IV) and Au(III) by Vonnel-en may depend on the sample volume and on the analyte concentration. These results are inconsistent with our previous experience of the solid-phase extraction of metal ions, wherein chelating adsorbents that have groups, such as 8-hydroxyquinoline [29] and ethylenediaminetriacetic acids, were used [30]. In these studies, metal ions were quantitatively recovered, independent of sample volume and metal ion concentrations, as long as the total amount of metal ions was sufficiently lower than the adsorption capacity of the chelating column. A possible explanation is that en is a neutral ligand and forms less stable chelate with metal ions compared with negatively charged ligands. The reason of difference between TYP-en and Vonnel-en is not clear now. It is possible that the nature of copolymer of Vonnel-en causes the difference.

## Conclusions

In this study, a chelating fiber Vonnel-en and a chelating resin TYP-en, which have en groups, were synthesized to evaluate their performance in a solid-phase extraction of Pd, Pt, and Au. In batch adsorption experiments, the adsorption capacity of Vonnel-en was 1.7 and 1.3 times higher than that of TYP-en for Pd(II) and Pt(IV), respectively, whereas the adsorption capacity of Vonnel-en was half that of TYP-en for Au(III). Column extraction experiments revealed that TYP-en is superior to Vonnel-en. In experiments using small-volume samples containing a metal ion at a µmol/kg concentration, Vonnel-en could not quantitatively recover Au(I) from 0.07 to 0.2 M HCl solution. However, TYP-en was able to quantitatively recover Pd(II), Pt(II), Pt(IV), Au(I), and Au(III) from 0.01 to 0.2 M HCl solution. In experiments using large-volume samples containing metal ions at pmol/kg concentrations, Vonnel-en was unable to quantitatively recover Pt(IV) and Au(III). However, TYP-en could quantitatively recover Pd(II), Pt(IV), and Au(III) simultaneously. Based on these results, TYP-en was concluded as a more promising adsorbent than Vonnel-en for the preconcentration of Pd, Pt, and Au. As the en groups both participate in anion exchange and chelate formation, TYP-en is effective for the solid-phase extraction of Pd, Pt, and Au chloride complexes. Future investigations will include the development of an analytical method for the determination of Pd, Pt, and Au in environmental water samples, such as river water and seawater.

## Supplementary Information

Below is the link to the electronic supplementary material.

Supplementary file1 (DOCX 135 KB)

## Data Availability

All data generated or analyzed during this study are included in this published article.
