# Supplementary material for: Solid-phase extraction of palladium, platinum, and gold from water samples: comparison between a chelating resin and a chelating fiber with ethylenediamine groups
Source: Anal Sci. 2023 Jan 19;39(5):695–704. doi: 10.1007/s44211-023-00270-3 (PMC10121507; doi:10.1007/s44211-023-00270-3)
Supplement: Supplementary file 1 — Supplementary file1 (DOCX 135 KB) [file 44211_2023_270_MOESM1_ESM.docx]

Supplementary Information

Solid-phase extraction of palladium, platinum, and gold from water samples: comparison between a chelating resin and a chelating fiber with ethylenediamine groups

Misato IWASE^†^, Kota ISOBE, Linjie ZHENG, Shotaro TAKANO, and Yoshiki SOHRIN

*Institute for Chemical Research, Kyoto University, Uji, Kyoto 611-0011, Japan*

^†^To whom correspondence should be addressed.

iwase.misato.6v@kyoto-u.ac.jp

1. Data on the optimization of eluents


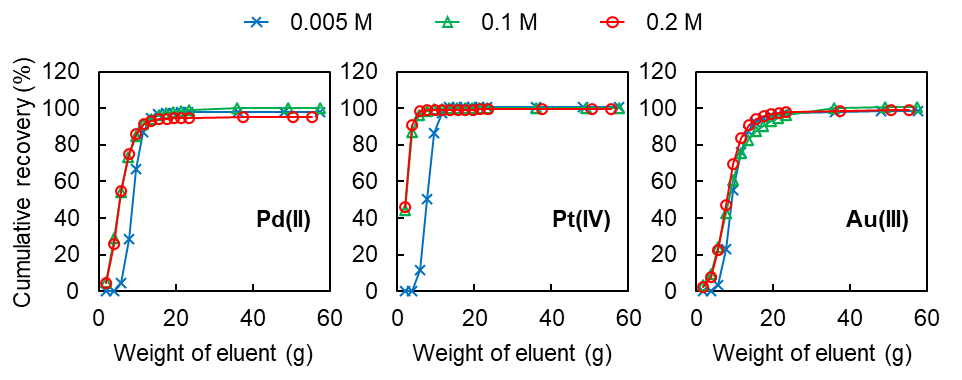


**Fig. S1** Effect of NH_3_ concentration and weight of eluent on the recovery of Pd(II), Pt(IV), and Au(III) by column extraction with TYP-en. Sample solution: 25 g of 0.1 M HCl solution containing 50 μmol/kg Pd(II), 25 μmol/kg Pt(IV), or 25 μmol/kg Au(III). Eluent: 0.005–0.2 M NH_3_–0.001 M KCN (*n* = 1).


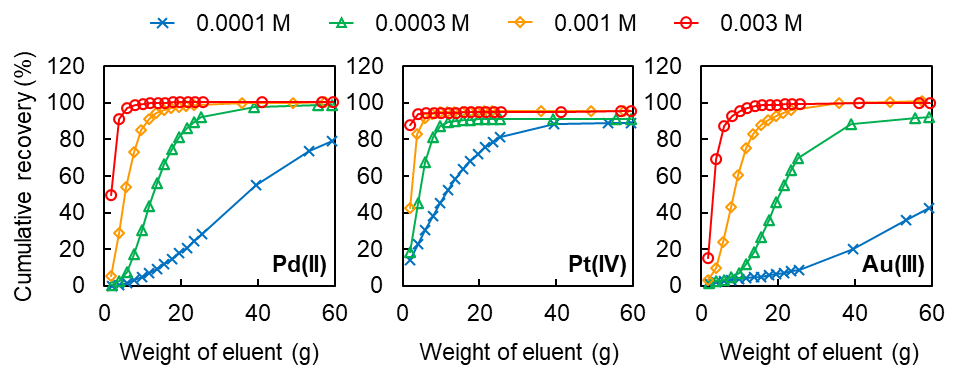


**Fig. S2** Effect of KCN concentration and weight of eluent on the recovery of Pd(II), Pt(IV), and Au(III) by column extraction with TYP-en. Sample solution: 25 g of 0.1 M HCl solution containing 50 μmol/kg Pd(II), 25 μmol/kg Pt(IV), or 25 μmol/kg Au(III). Eluent: 0.1 M NH_3_–0.0001–0.003 M KCN (*n* = 1).


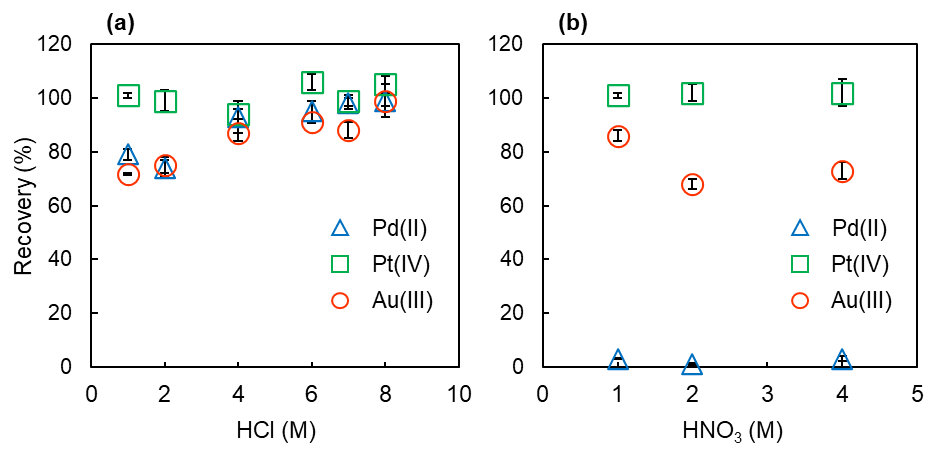


**Fig. S3** Effect of (a) HCl or (b) HNO_3_ concentration of eluent on the recovery percentage of Pd(II), Pt(IV), and Au(III) by column extraction with TYP-en. For elution with 1–2 M HCl and 1–4 M HNO_3_, the samples used were 25 g of 0.07 M HCl containing 50 μmol/kg Pd(II), 25 μmol/kg Pt(IV), and 25 μmol/kg Au(III). For elution with 4–8 M HCl, the samples used were 25 g of seawater at pH 1.55 containing 50 μmol/kg Pd(II), 25 μmol/kg Pt(IV), and 25 μmol/kg Au(III). The amount of eluent was 90 g. Error bars show the standard deviation (*n* = 3 for 1–2 M HCl and 1–4 M HNO_3_, *n* = 2 for 4–8 M HCl).

1. Data on the effect of matrixes in river water

Table S1 Concentrations of elements in eluates after the column extraction of Pd, Pt, and Au by TYP-en from a spiked river water.

| Eluent | Metal amount in eluate | | | | |
| --- | --- | --- | --- | --- | --- |
|  | Fe (nmol) | Mn (nmol) | Pd (pmol) | Pt (pmol) | Au (pmol) |
|  | ave ± sd | ave ± sd | ave ± sd | ave ± sd | ave ± sd |
| 0.3 M NH_3_–0.003 M KCN | not measured | not measured | 1.2 ± 0.1 | 0.11 ± 0.01 | 2.1 ± 0.1 |
| 1 M HCl | 2.7 ± 0.2 | 0.13 ± 0.01 | 0.9 ± 0.3 | 0.12 ± 0.16 | 1.4 ± 0.4 |
| 1 M HNO_3_ | 0.4 ± 0.1 | 0.02 ± 0.01 | 0.8 ± 0.1 | 0.25 ± 0.15 | 2.7 ± 0.2 |

The sample was collected from the Uji River, filtered with a Sterivex filter unit with a pore size of 0.22 µm (Merck), and acidified to 0.07 M HCl. A portion of 607 g of the river water sample was spiked with 3.25 pmol Pd, 3.22 pmol Pt, and 3.21 pmol Au, which was applied for the column extraction with TYP-en in the same manner with that described in the manuscript. Then the metal ions were eluted successively with 60 g of 0.3 M NH_3_–0.003 M KCN, 60 g of 1 M HCl, and 60 g of 1 M HNO_3_. The concentrations of Fe, Mn, Pd, Pt, and Au in eluates were measured by ICP-MS (*n* = 2).

The data indicate that some parts of Fe and Mn in the river water were collected on TYP-en and eluted with 1 M HCl and 1 M HNO_3_. Some parts of Pd, Pt, and Au were not eluted with 0.3 M NH_3_–0.003 M KCN and were eluted with acid solutions concurrently with Fe and Mn.
